# Mapping of 10-km daily diffuse solar radiation across China from reanalysis data and a Machine-Learning method

**DOI:** 10.1038/s41597-024-03609-1

**Published:** 2024-07-11

**Authors:** Qinghai Qi, Jinyang Wu, Christran A. Gueymard, Wenmin Qin, Lunche Wang, Zhigao Zhou, Jiayun Niu, Ming Zhang

**Affiliations:** 1 Hubei Key Laboratory of Regional Ecology and Environment Change, School of Geography and Information Engineering, China University of Geosciences, Wuhan, 430074 China; 2 Solar Consulting Services, Colebrook, NH USA; 3 School of Low Carbon Economics, Hubei University of Economics, Wuhan, 430074 China

**Keywords:** Atmospheric dynamics, Solar thermal energy

## Abstract

Diffuse solar radiation (DSR) plays a critical role in renewable energy utilization and efficient agricultural production. However, there is a scarcity of high-precision, long-term, and spatially continuous datasets for DSR in the world, and particularly in China. To address this gap, a 41-year (1982–2022) daily diffuse solar radiation dataset (CHDSR) is constructed with a spatial resolution of 10 km, based on a new ensemble model that combines the clear-sky irradiance estimated by the REST2 model and a machine-learning technique using precise cloud information derived from reanalysis data. Validation against ground-based measurements indicates strong performance of the new hybrid model, with a correlation coefficient, root mean square error and mean bias error (MBE) of 0.94, 13.9 W m^−2^ and −0.49 W m^−2^, respectively. The CHDSR dataset shows good spatial and temporal continuity over the time horizon from 1982 to 2022, with a multi-year mean value of 74.51 W m^−2^. This dataset is now freely available on figshare to the potential benefit of any analytical work in solar energy, agriculture, climate change, etc (10.6084/m9.figshare.21763223.v3).

## Background & Summary

Diffuse solar irradiance (DSR) is a critical component of solar radiation, substantially influenced by atmospheric conditions such as aerosols and cloud cover^1,2^. Accurately modeling DSR is crucial not only for agriculture applications, where it enhances plant productivity and photosynthetic efficiency by modifying the light environment, but also for border environment and economic impacts^3,4^. Variability in DSR predictions can significantly affect climate modeling and carbon budget assessments, potentially leading to large discrepancies in environment policy and climate strategy effectiveness. Moreover, as global efforts intensify to achieve carbon neutrality for the strategic placement and efficiency optimization of solar power systems^5^. Therefore, improving the accuracy of DSR predication is essential, not only for advancing agriculture productivity but also for enhancing the reliability of renewable energy resources and supporting robust climate change responses^6,7^.

Observations from accurately-calibrated and continuously-serviced pyranometers are the most effective way to obtain reliable, long-term DSR data. However, considering the construction and maintenance costs, out of the 119 stations operated by the China Meteorological Administration (CMA), only 17 of these CMA stations are equipped for the monitoring of DSR. In contrast, satellite remote sensing can be used to derive spatiotemporally continuous estimates of surface solar irradiance on a regional or global scale^8–13^. Satellite-based retrieval models can be classified into two categories: semi-empirical models^14^ and physical model^15,16^. Semi-empirical models first estimate the surface global horizontal irradiance (GHI), then one empirical model (among many possibilities^17^) is operated to separate it into its direct and diffuse components. However, the actual performance of these empirical models is generally station dependent^18^, which can lead to large deviations unless a local post-processing adjustment (usually referred to as “site adaptation”) is carried out^19,20^. Yu^21^ used an improved separation model while introducing the *Kt*-*K* group criterion, which resulted in hourly DSR predictions having a relative root mean square error (rRMSE) of ≈26–44% at three control stations.

Physical radiative transfer models have been applied to estimate solar radiation around the world^22–24^, but such models are too complex and slow to be used in operational satellite retrievals. Simpler parameterized models are thus rather utilized for such tasks, in particular to obtain the irradiance components under ideal cloud-free conditions. Such models can be divided into the spectral^25^ and broadband^1^ types. Simplified physical models like Solar Irradiance Scheme (SOLIS) or Fast All-sky Radiation Model for Solar applications with Narrowband Irradiances on Tilted surface (FARMS-NIT) can provide spectrally resolved irradiance data under clear-sky conditions and/or cloudy conditions^26–28^, using satellite-derived information. In parallel, the REST2 model (Reference Evaluation of Solar Transmittance, 2 bands) can estimate the broadband surface irradiance with similar accuracy as more sophisticated spectral models, yet with much lower computational requirements^29^, and thus has been widely used for estimating global, direct, and diffuse solar radiation.

Under some cloudy and/or humid weather conditions, such as in tropical climates, the quality of satellite remote-sensing observations can be limited, which constrains the accuracy of physical models that require high-precision cloud optical property data. Compared to the loss of accuracy caused by the noisy environment for conventional radiation models, machine learning (ML) algorithms are good candidates to achieve good DSR estimates under cloudy and/or hazy conditions, even with limited cloud and aerosol optical properties as input variables. Various ML methods have been developed to improve prediction accuracy in cloudy environments, including Support Vector Machine (SVM), Random Forest (RF), Artificial Neural Networks (ANN) and Deep Neural Networks (DNN)^30–34^. For example, Shamshiband *et al*.^35^ proposed a hybrid model by integrating SVM with the Wavelet Transform algorithm. Their results demonstrate that this hybrid model yields good predictions and offers significantly higher accuracy than SVM or ANN. Fan *et al*.^36^ generated three new hybrid SVM models with heuristic algorithms, compared to the stand-alone SVM model, the hybrid model performed more precisely and reliably. Wu *et al*.^37^ generated a high resolution DSR datasets based on a generalized additive models (GAM) that cooperates with several ML models. Zhao *et al*.^38^ established a hybrid model based on four XGB boosting models to improve the satellite-based DSR products.

The existing literature shows that the key role that can be played by ML in tackling cloud uncertainty remains undiminished, despite the challenges^39,40^. On this basis, various DSR datasets have been prepared based on through ML methods under all-sky conditions. For example, Jiang^15^ produced a 12-year (2007–2018) hourly solar radiation dataset at 5-km resolution based on DNN. In parallel, Chakraborty^41^ used RF algorithms to generate a 40-year (1980–2019) monthly dataset using information from NASA’s MERRA-2 reanalysis, with a spatial resolution of 0.5 × 0.625°. Both have advantages in terms of the spatial and temporal resolution as well as spatial/temporal extent, and disadvantages in terms of each other’s strengths. In addition, well-established databases such as ERA5, SARAH-E, or CERES all supply DSR datasets at different scales. However, these DSR products are subject to large uncertainties, at least over China. For instance, the RMSE and rRMSE of the daily mean DSR obtained by ERA5 and CERES products under all-weather conditions were found to be above 30 W m^−2^ and 40%, respectively^37,42^, while the rRMSE for SARAH was even above 50%.

At global or continental spatial scale, the existing DSR datasets lack simultaneous high spatial resolution and temporal continuity. In this study, the REST2 radiation model is used to generate a DSR dataset at high spatial resolution under clear-sky conditions. Subsequently, a novel hybrid model that combines the REST2 clear-sky estimates with a ML stacking model is developed to construct a continuous and gridded DSR dataset with a high resolution of 10 km over China, and a long span of 41 years (1982–2022).

## Methods

### Validation data

**Fig. 1:**
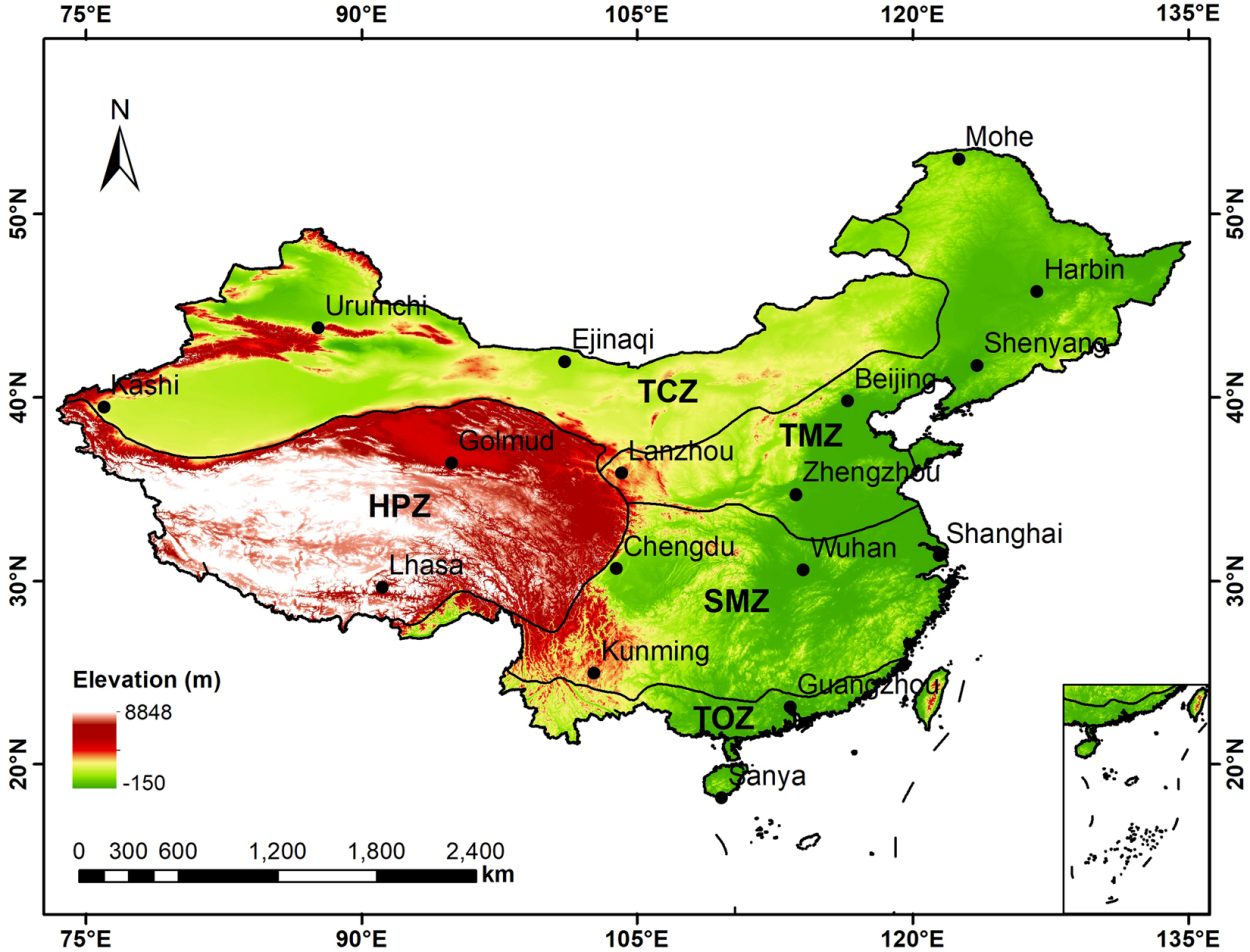
Spatial distribution of 17 CMA radiometric stations that observe DSR over China, superimposed with a digital elevation model.

The surface DSR observations used in this study are recorded at 17 stations maintained by CMA. The 2011–2015 data from all these stations are used for the training states of the hybrid ML model, whereas the 2010 data are used for independent validation. Moreover, the daily DSR records from the same sites but for the 2000–2015 extended period are used for the validation of the modeled DSR values across the country’s five major climate zones. Temperate continental zone (TCZ), Temperate monsoon zone (TMS), High-mountain plateau zone (HPZ), Subtropical monsoon zone (SMZ), and Tropical monsoon zone (TOZ). Figure 1 shows the spatial distribution of the 17 radiometric sites over China, superimposed with an elevation map of the country.

The ground-based DSR observations used in this study are obtained with pyranometers that were developed by CMA. A shade disk is attached to a solar tracker to block the direct radiation from the pyranometer and only sense DSR. The quality-control method of the measured DSR data includes three successive steps: (1) Climate-dependent thresholding and permissible value check^43^; (2) Internal consistency check; and (3) Time continuity check.

### Reanalysis data

The European Centre for Medium-Range Weather Forecasts (ECMWF) has developed the ERA5 long-term reanalysis dataset, providing several products with various spatial and temporal resolutions^44^. The main ERA5 reanalysis provides meteorological data at a single levels or at the surface, including ozone, water vapor, etc., with a spatial resolution of 0.25° × 0.25° and a temporal resolution of 1 h since 1940^45^. In what follows, it is referred to as ERA5-Single. In parallel, the ERA5-Land dataset, which starts only in 1950, has a finer resolution of 0.1° × 0.1°^46^. In addition, NASA’s Modern-Era Retrospective analysis for Research and Applications, Version 2 (MERRA2) reanalysis also provides some additional inputs for the hybrid model^47^. MERRA-2 products are widely used in solar applications (among others) because of their excellent spatial and temporal continuity^48,49^. In particular, its aerosol optical depth (AOD) estimates are considered of high quality^50^ and are frequently used for solar irradiance modeling^11,51^. Details on the reanalysis data used in this study are given in Table 1.

**Table 1:** Information on the input variables obtained from reanalysis datasets.

Dataset	Parameter Used in This Work	Spatial Resolution	Temporal Resolution
ERA5-Single	Total Column Ozone (TCO), Total Column Water Vapor (TCWV), High Cloud Cover (HCC), Medium Cloud Cover (MCC), Lower Cloud Cover (LCC), Boundary Layer Height (BLH), Total Cloud Cover (TCC), Total Column Cloud Ice Water (TCCIW), Total Column Cloud Liquid Water (TCCLW), Top-of-atmosphere Incident Solar Radiation (TISR)	0.25° × 0.25°	Hourly
ERA5-Land	Forecast Albedo (FA), Surface Pressure (SP), Surface Solar Radiation Downwards (SSRD), 2 m Temperature (T2m), Total Precipitation (TP)	0.1° × 0.1°	Hourly
MERRA-2	Aerosol Optical Depth (AOD) at 550 nm	0.5° × 0.625°	Hourly

### Clear-sky diffuse irradiance estimation

In this study, the REST2 model^1^ provides estimates of DSR under clear-sky conditions. REST2 consists of parameterizations of look-up tables obtained from the SMARTS spectral model^25,52^. The different sources of atmospheric attenuation are parameterized for each of two spectral bands (0.28–0.7 µm and 0.7–4.0 µm). The model has been thoroughly validated against various sources of high-quality irradiance data^29,53,54^. Version 9.1 of the REST2 model is used here; it incorporates a number of improvements (particularly related to the modeling of diffuse irradiance) compared to the publicly-available version 5^55^. For the present application, the input variables are local standard time (Year, Month, Day, Hour), surface pressure, regional ground albedo, reduced ozone vertical pathlength precipitable water, and AOD at 550 nm. These variables are uniformly interpolated from their ERA5 or MERRA-2 sources to a uniform resolution of 0.1° × 0.1°, and then applied as input variables to REST2 to generate 41 years of hourly clear-sky DSR data for every single pixel over China.

Under clear-sky conditions, the REST2 derived DSR maps produced here for China can be considered of high quality. In turn, these clear-sky DSR maps can be used as the necessary foundation to apply the machine-learning algorithm that evaluates the cloud impacts, as explained below, and ultimately to prepare maps at relatively high spatial resolution.

### All-sky diffuse solar radiation estimation

After estimating the REST2 estimates of clear-sky diffuse solar radiation, the results are combined with various cloud properties derived from ERA5 to form the input data to the ML model that is tasked to estimate DSR under all-sky conditions. Meanwhile, it should be noted that since the model is constructed based on the station observation data from CMA, but since we lack hourly-scale station observation data it is difficult to obtain the data in China at an hourly scale, we set both the output and the input of the all-sky model as daily scale. In selecting the input variables, we relied heavily on guidance from previous literature and the availability of data^56^. Based on previous studies, it was possible to ensure that the variables selected could represent the combined effects of different meteorological and geographical factors on solar radiation.

**Fig. 2:**
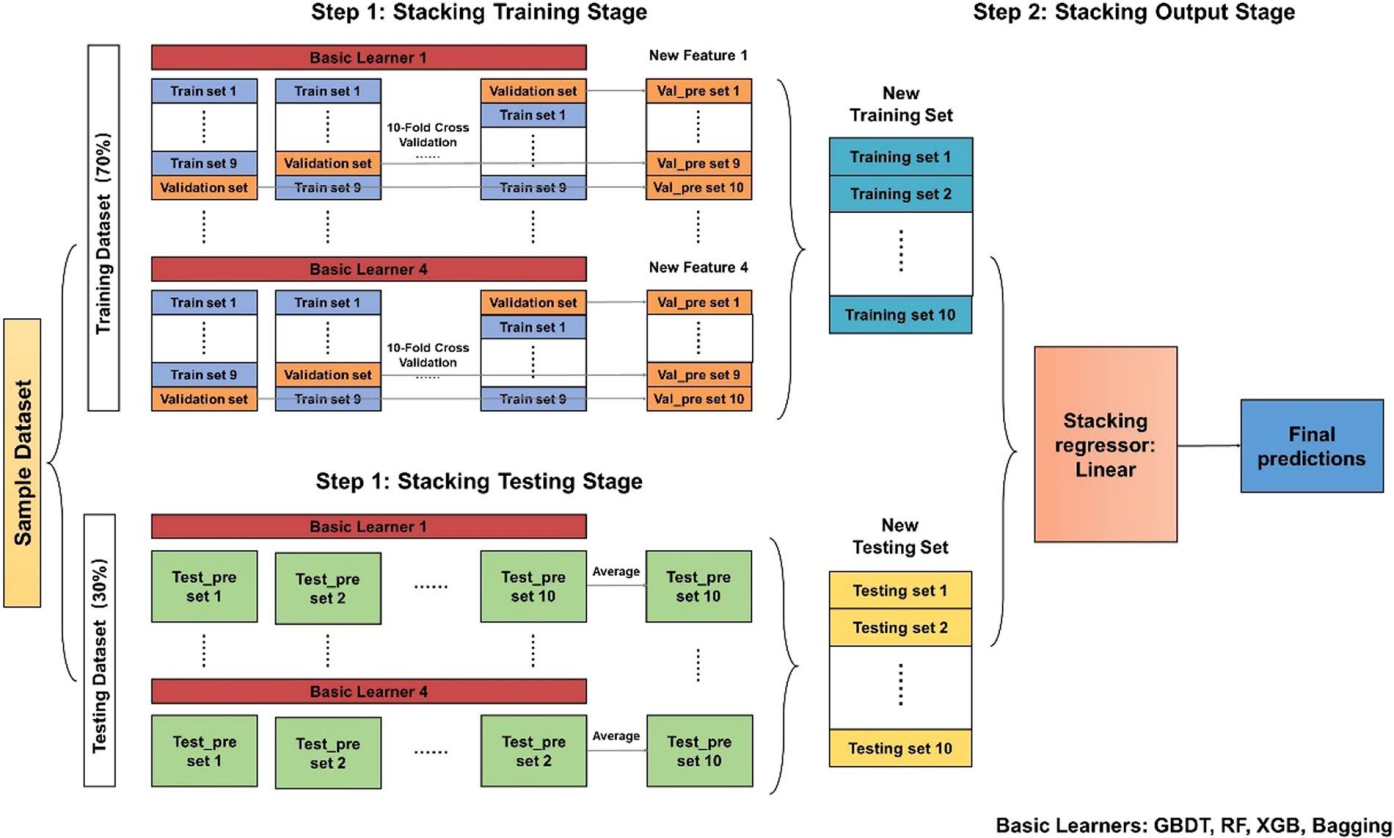
Schematic flowchart used to generate diffuse solar radiation datasets.

The process of constructing the DSR estimation model based on the stacking method is shown in Fig. 2. In this study, six years of data samples from 2011–2015, totaling 36492 entries, are formed based on the matching records at 17 CMA stations. Each base learner (GBDT, RF, XGB, Bagging) builds a non-linear relationship between the input variables and the DSR results with the same training set. Then, the base learner, which constitutes the ensemble learning, builds an improved DSR estimation result.

All data sets are constructed with a 7:3 ratio of training and test sets, and the ten-fold cross-validation is performed on the training data to distinguish the sub-training set and sub-validation sets in the training set. The training and validation sets of the meta-learner are obtained in the form of weighted averages. In this study, the correlation coefficient R, the mean bias error (MBE), the root-mean square error (RMSE) and the relative root-mean square error (rRMSE) are used to quantify the quality of the estimation results.

The main concept behind integrated modelling is to combine multiple base models to create a more comprehensive and robust model for strongly supervised learning. By appropriately combining these base models, a reduction in variance, bias, or improved prediction can be achieved. One approach, known as the stacking model, allows the use of different types of models as primary learners. This involves aggregating the outputs of these base learners to form the final output, while training the primary learners independently. The meta-models are then trained using the outputs of the primary learners as inputs and the output data of the training set. In parallel, the GridSearchCV method is used to optimize the hyperparameters of the ML model. The main steps include: (i) separating the training and test samples; (ii) identifying the parameters to be optimized and setting the others to default values; (iii) adjusting them sequentially in steps within the specified parameter range; and (iv) iterating the paths. This whole process result in what is referred to below as “stacking model”.

### Validation of modelled DSR using different clear-sky detection methods

The REST2 model is designed to estimate solar radiation under cloudless conditions, which means that the choice of an appropriate clear-sky detection (CSD) method is important for evaluating the accuracy of such predictions. These methods attempt to detect clear-sky periods based on an analysis of historical time series of at least one component of solar irradiance. Many methods have been proposed in the literature, and the typically provide different (more or less stringent) results, as reviewed in^57^. Here, three different clear-sky detection methods of various complexity and requirements are tested:

- CSD1: Simple method uniquely using the clearness-index, *Kt*, defined as the ratio between GHI and its extraterrestrial counterpart; this method has been popular in earlier solar resource studies.
- CSD2: Clear-sky detection method according to Long & Ackerman^58^; this method has been widely used by the atmospheric sciences community.
- CSD3: The BrightSun method, which is more elaborate and the most recent detection method.

**Fig. 3:**
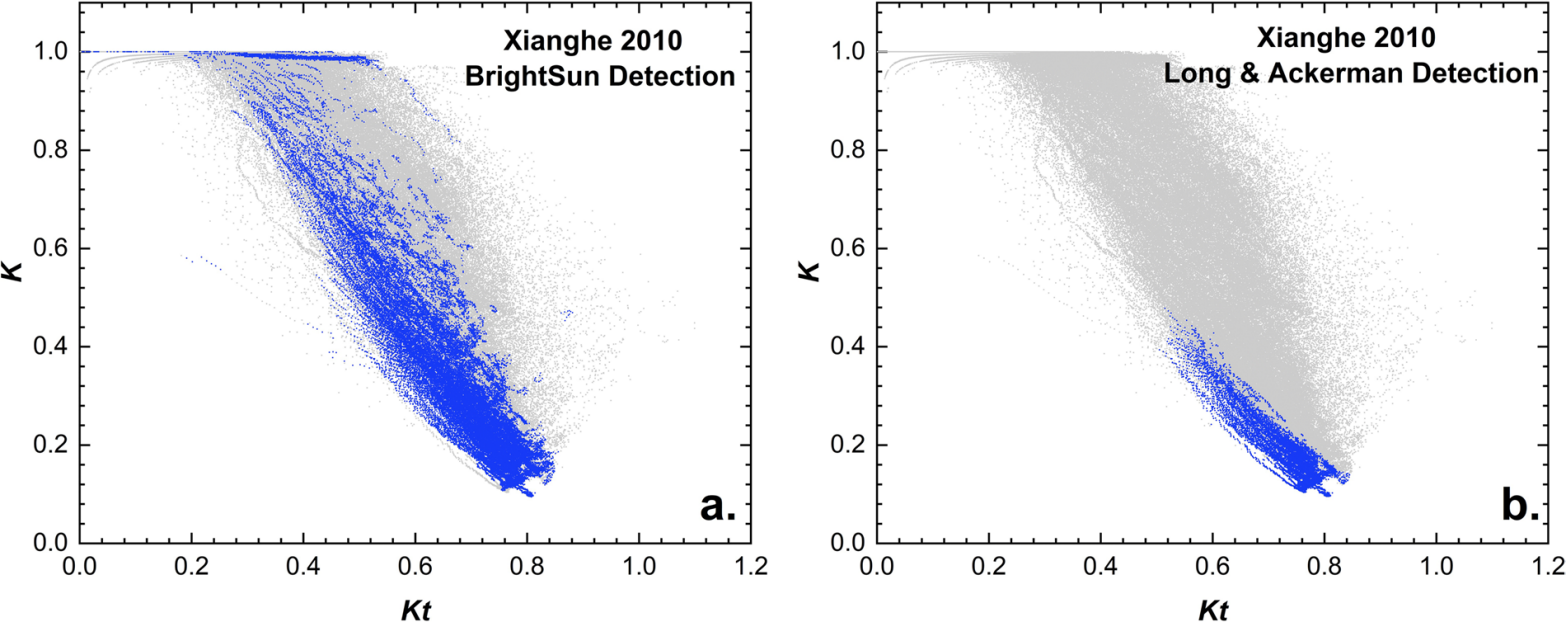
Comparison of the CSD2 and CSD3 clear-sky detection methods at the Xianghe BSRN station in 2010. *K* is the diffuse fraction, i.e., the ratio between DSR and GHI. Blue dots represent the clear-sky detection results using two different detection methods: (**a**). Long & Ackerman (CSD2) and (**b**). BrightSun (CSD3).

The Chinese city of Xianghe harbored one radiometric station of the BSRN network until 2014^59^. The station provided observations of the three radiation components at 1-min resolution, and is thus qualified to test even the most demanding CSD methods, such as CSD2 or CSD3. In the literature, it is often considered that clear-sky situations occur when the clearness index is larger than 0.7^60^. Comparatively to that simple (and questionable) assumption, the Long & Ackerman method has a more physical background, whereas BrightSun represents an even more advanced methodology, based on modifications to the Reno-Hansen method^61^. Whereas the latter only requires GHI measurements, BrightSun also depends on those of either direct or diffuse irradiance, just like Long & Ackerman. Figure 3 compares the 1-min clear-sky periods detected by CSD2 and CSD3 at Xianghe in 2010. These results are plotted in *K*-*Kt* space, where *K* represents the diffuse fraction, i.e., DSR/GHI. It is clear that, under low *Kt* CSD3 detection returns more clear periods than CSD2, in part because the latter does not operate under low-sun conditions. This finding also means that there are limitations to the application of CSD1, since there appears to be many clear situations when *Kt* < 0.7, most likely because of the generally hazy conditions at Xianghe. Moreover, Fig. 3 indicates that CSD3 effectively filters out situations when various types of clouds have a small effect on GHI but a large effect on DSR. A general limitation of all simple CSD methods, however, is that they typically have difficulty detecting clear conditions under extremely hazy conditions, which do occur in Xianghe. In that sense, CSD3 does appear to detect many such clear but hazy situations, whereas CSD2 appears much too stringent. An important consequence of these findings is that the validation of clear-sky radiation models, such as REST2, would not be possible under historical high-turbidity conditions if using CSD1 or CSD2. Conversely, some of the scenes reported as clear by CSD3 could actually be cloudy.

**Fig. 4:**
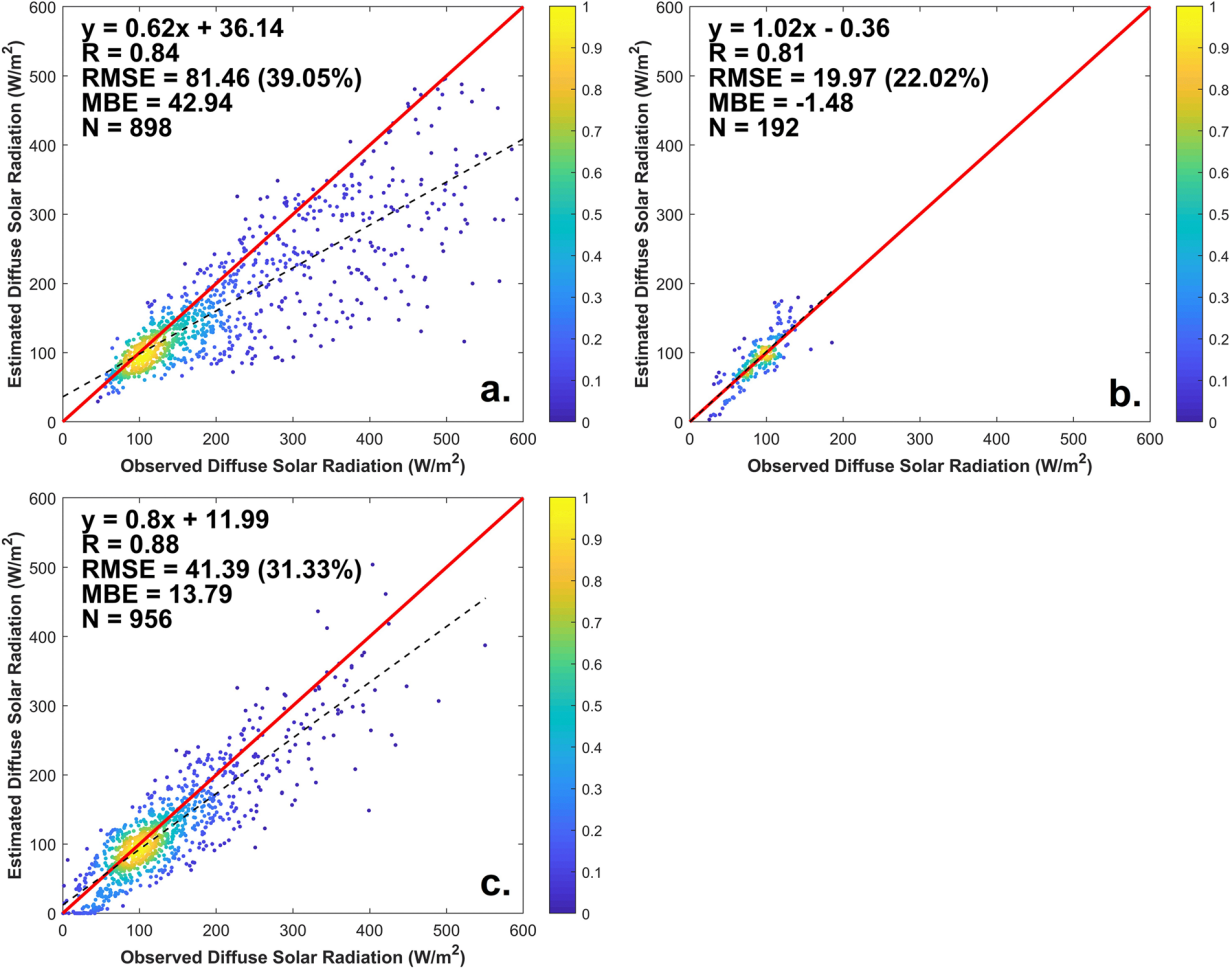
Comparison between REST2 estimates of DSR and their measured counterpart at Xianghe under the clear-sky conditions detected by three different clear-sky detection methods in 2010: (**a**) CSD1;(**b**) CSD2 and (**c**) CSD3.

Figure 4 shows how the REST2 hourly predictions compare to the DSR observations at Xianghe in 2010, alternatively using the three CSD methods. Overall, REST2 demonstrates excellent accuracy in estimating the hourly DSR under clear-sky conditions, with a correlation coefficient above 0.84 and a RMS error of 81.5 W m^−2^ (39.1%) for CSD1 (N = 898), 20.0 W m^−2^ (22.0%) for CSD2 (N = 192), and 41.4 W m^−2^, (31.3%) for CSD3(N = 956). Figure 4 shows that the DSR results are concentrated around ≈100 W m^−2^, while hourly clear-sky DSR observations can reach ≈600 W m^−2^, i.e., very hazy conditions, using either CSD1 or CSD3. In contrast, CSD2 can only detect clear periods with DSR up to only ≈180 W m^−2^, thus excluding the haziest conditions, as discussed above. BrightSun appears the most appropriate CSD method in a hazy environment such as Xianghe, or many other urban areas in China. In all plots of Fig. 4, the remaining scatter can be explained by imperfections in the CSD method and/or by imperfections in the critical inputs to the model, particularly in terms of AOD. In conclusion, the REST2 estimation of DSR under clear-sky conditions are found reliable overall, thus providing a stable foundation for in the ML modeling step, which is necessary to estimate the all-sky irradiance.

## Data Records

Using the models described above, a database of daily average all-sky DSR, referred to as CHDSR^62^, has been produced for China. This database covers the 41-year period from 1982 to 2022, and can be downloaded from a dedicated website (10.6084/m9.figshare.21763223.v3). The name of each data file is formatted as “DIF_yyyy.nc”, where “yyyy” represents the year, and the diffuse solar radiation is stored in the netCDF file as floating-point values in “W m^−2^”. Each file contains three variables (DSR, Longitude, and Latitude), with dimensions of 641 × 361. The latitude and longitude ranges covered by this dataset are 72°–136°E, 18°–54°N, respectively, with a spatial resolution of 0.1° (≈10 km); the time format used is local standard time (China’s time zone is +8).

## Technical Validation

### Validation against ground measurements

**Fig. 5:**
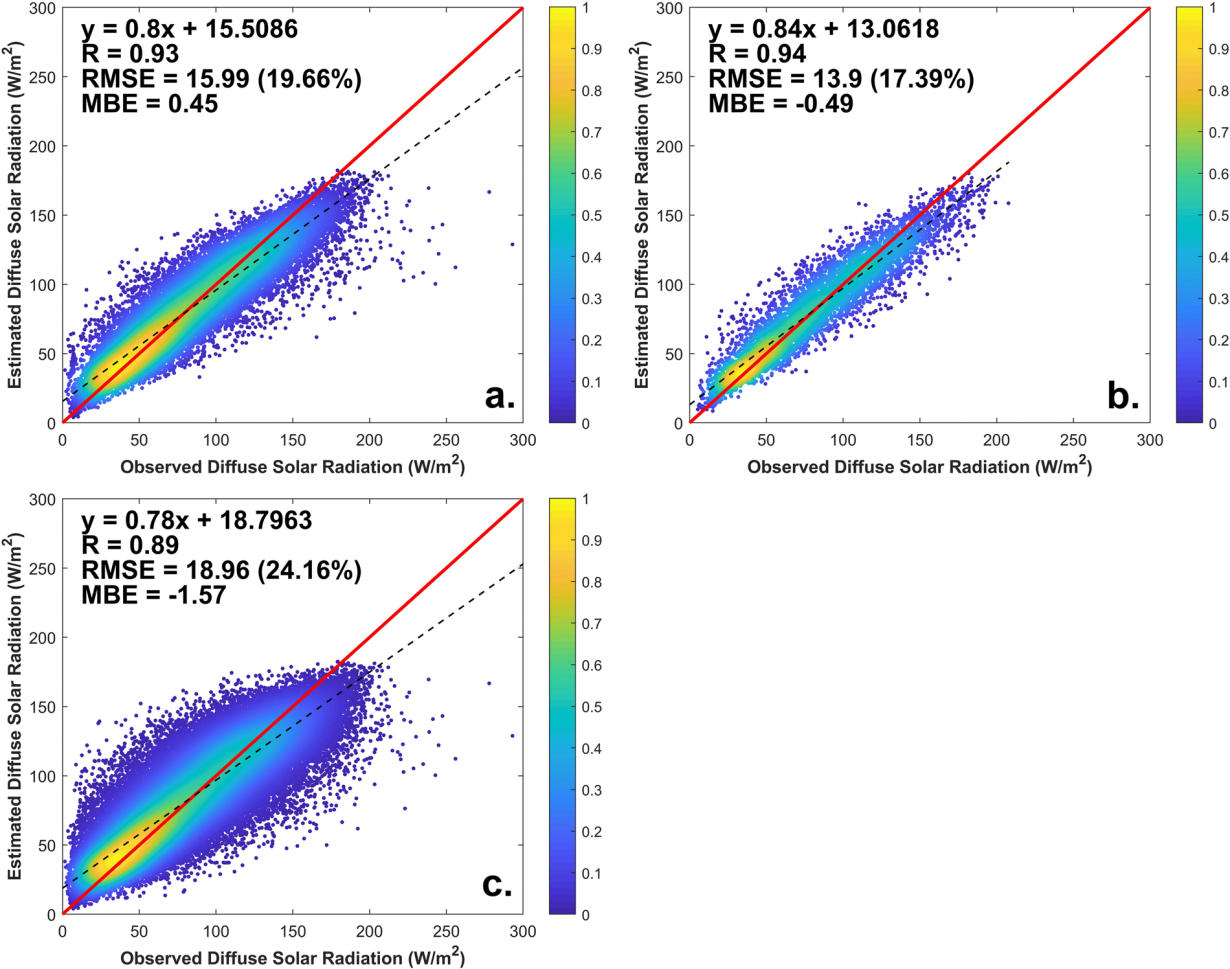
Density scatterplots of the CHDSR modeled dataset at daily resolution compared to measured data from 17 test stations. (**a**) Evaluation during 2011–2015; (**b**) Evaluation for an independent year, 2010; and (**c**) Evaluation during 2000–2015.

The performance of the stacking model for the CHDSR^62^ dataset is evaluated to check the viability of long-term estimation. Firstly, the model training set is validated for the period 2011–2015, as shown in Fig. 5a. The test set for 2010 is also validated, as depicted in Fig. 5b. Additionally, the extended 2000–2015 time range also is finally validated, as illustrated in Fig. 5c. Overall, the stacking model performs well against the test set from 2000 to 2015, with R values ranging from 0.89 to 0.94, RMSE values ranging from 13.9 to 19.0 W m^−2^, and MBE values ranging from −1.6 to 0.5 W m^−2^. The sample-based cross validation R, RMSE and MBE results are 0.93, 16.0 W m^−2^, 0.5 W m^−2^, respectively. Using the test set of 2010, the stacking model performs better, with an R value of 0.94, RMSE of 13.9 W m^−2^, and MBE of −0.5 W m^−2^. The deviation of the stacking model remains low for both the training and test sets, showing the stability of the model’s estimation results. Furthermore, over the extended 2000–2015 time period, the model retains high accuracy with R = 0.89, RMSE = 19.0 W m^−2^, and MBE = −1.6 W m^−2^, thus revealing the powerful temporal extensibility of the stacking model in estimating DSR. The low RMSE and MBE confirm that the proposed datasets have a high degree of accuracy over China, with the desired stability for long-term estimation.

The CHDSR^62^ also delivers the best results and lowest bias when compared to other DSR products. For example, ERA5 also provides DSR products, but a significant underestimation of approximately 43.1 W m^−2^ has been observed over China, apparently caused by poor estimates of the cloud path^63^. The remote-sensed CERES product performs somewhat better with R = 0.8 and RMSE = 3.6 W m^−2 ^^37^. Jiang *et al*. obtained a better result with the R and RMSE of 0.79 and 20.1 W m^−2^, respectively^64^. In parallel, Jiang *et al*.^15^ also produced another daily DSR dataset with R = 0.89 and RMSE = 58.3 W m^−2^, respectively^15^. Wu *et al*. produced a high-resolution daily DSR dataset with R = 0.87 and RMSE = 20.2 W m^−2^ ^37^.

**Fig. 6:**
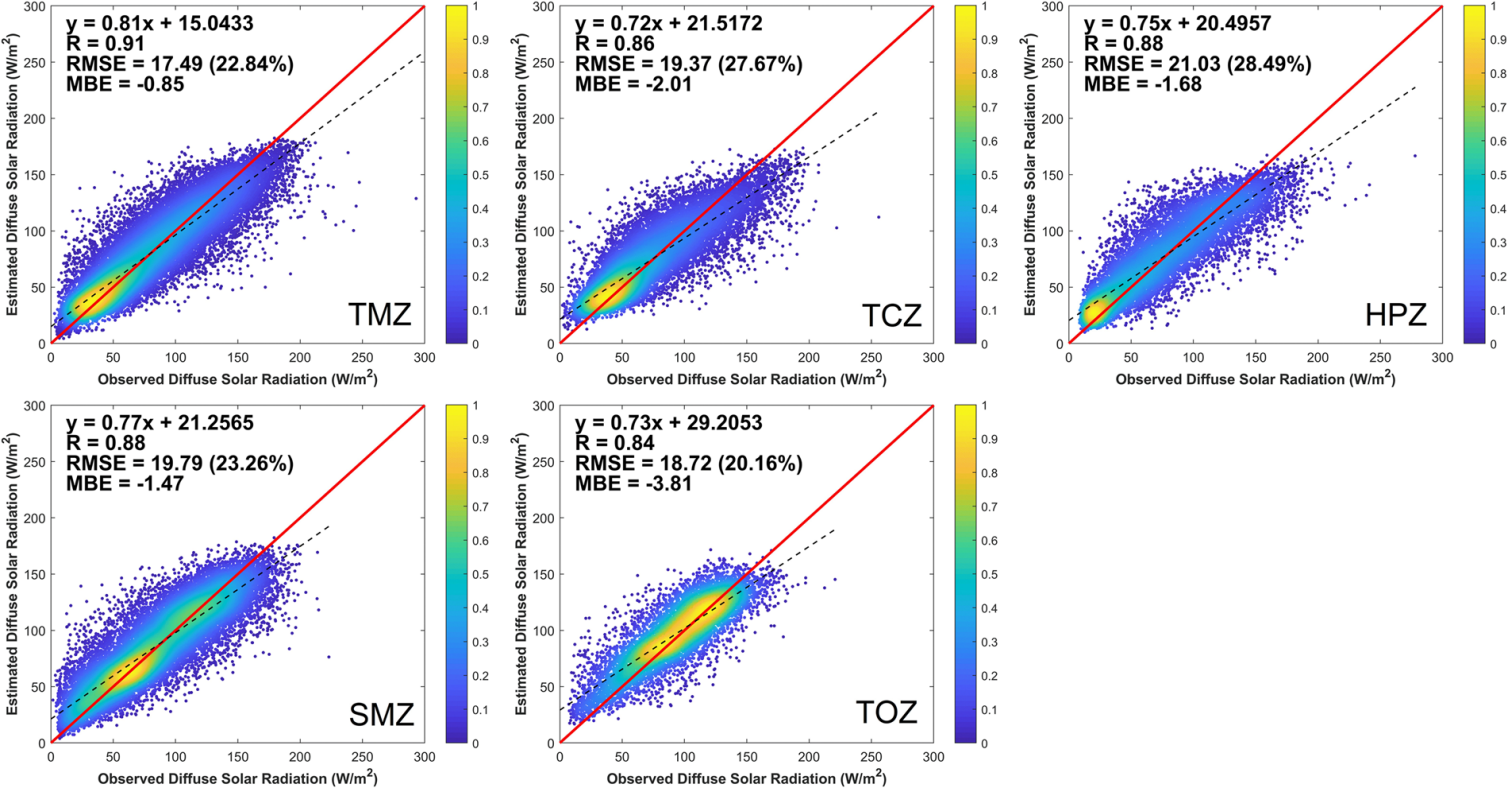
Density scatterplots of validation results for the five main climate zones of China.

To demonstrate the general applicability of the stacking model, the model is also validated separately for the five major climatic zones of China. The results displayed in Fig. 6 show that, in terms of R, the ranking is TMZ > SMZ > HPZ > TCZ > TOZ, all with rRMSE below 30%. Overall, the accuracy varies significantly over the different climate zones, confirming previous results^65^. For example, the modeled DSR performs best over TMZ with the highest R (0.91) and the lowest RMSE (18.72 W m^−2^), whereas the worst R (0.84) occurs for TOZ. One explanation is that the terrain is flat over TMZ, thus the remote-sensed or modeled input data (e.g., of AOD) are less affected by that important factors compared to the TOZ situation. An interesting observation is that the HPZ climate (which consists mainly of the Qinghai-Tibet Plateau) is the worst performing region in terms of RMSE, but not in terms of R. The largest scatter could be expected from the generally high elevations and substantial diversity in terrain features. In conclusion, the stacking model shows good applicability in the temperate monsoon climate zone, especially in the plains.

### Spatial distribution

**Fig. 7:**
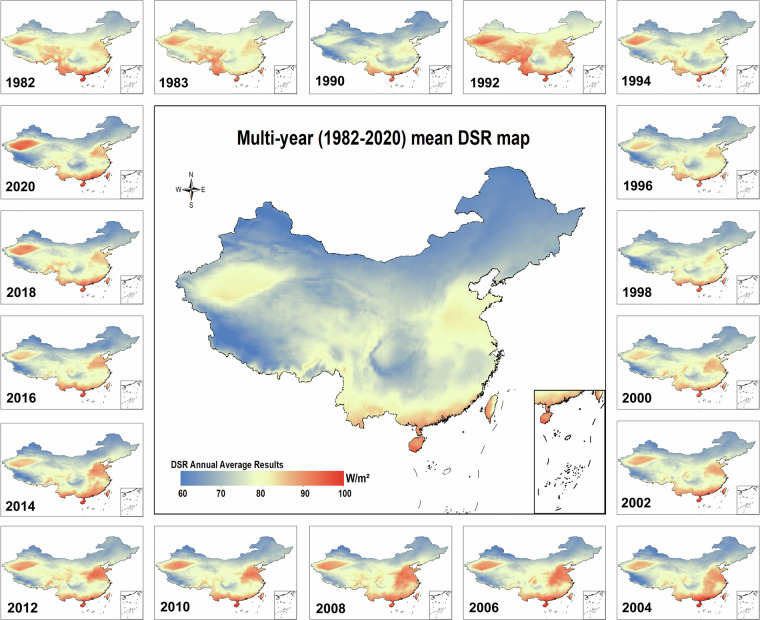
Annual and multi-year mean DSR over China with spatial resolution of 10 km for each year during 1982–2022.

Figure 7 illustrates the annual and multi-year mean DSR values for the period 1982–2020 over China. The diffuse solar radiation values range from 63.7 to 97.7 W m^−2^, with a 39-year average value of 77.0 W m^−2^. As depicted in the primary map in Fig. 7, elevated DSR values are predominantly concentrated in the Taklamakan Desert, Central and Southern China, and sections of the Qinghai-Tibet plateau. The presence of sand and dust aerosols amplifies the scattering effect, leading to elevated DSR values across the Taklamakan Desert^66^. The shorter radiation path in the Qinghai-Tibet region weakens the atmospheric molecular scattering effect, resulting in lower DSR values in that area. In contrast, the high levels of diffuse solar radiation over low-altitude and low-latitude areas, such as Guangzhou, can be attributed to a combination of increased industrialization and the abundance of sea salt aerosols from the monsoon^67,68^.

The mean diffuse solar radiation experienced significant turning points in 1990, 2000 and 2010, with DSR values of 75.6 W m^−2^, 78.8 W m^−2^,79.5 W m^−2^ respectively. However, in 2020, there was a slight decrease in the mean DSR (78.2 W m^−2^). At the insert of these four decades, the center of the low values was located in Inner Mongolia, with mean DSR of 63.8 W m^−2^, 68.5 W m^−2^, 72.3 W m^−2^, and 68.0 W m^−2^, respectively. This is mainly because of that area’s high geographical latitude, low sun elevation, and low aerosol burden, resulting in less solar radiation being scattered by the atmosphere. In 1990 and 2000, the centers of the high values were observed over high-altitude areas, specifically in Kunming. There, the mean DSR was 84.4 W m^−2^ during those years. This is attributed to the higher elevation of the Yunnan Plateau region, which was accompanied an increase in atmospheric transparency during the period, resulting in a brightening of the Southwestern region^69^. However, in 2010 and 2020, the center of the high DSR values moved to eastern China, an area characterized by significant urban and industrial developments and high anthropogenic pollutant emissions, resulting in mean DSR values of 92.2 W m^−2^ and 86.2 W m^−2^, respectively.

### Long-term trends

**Fig. 8:**
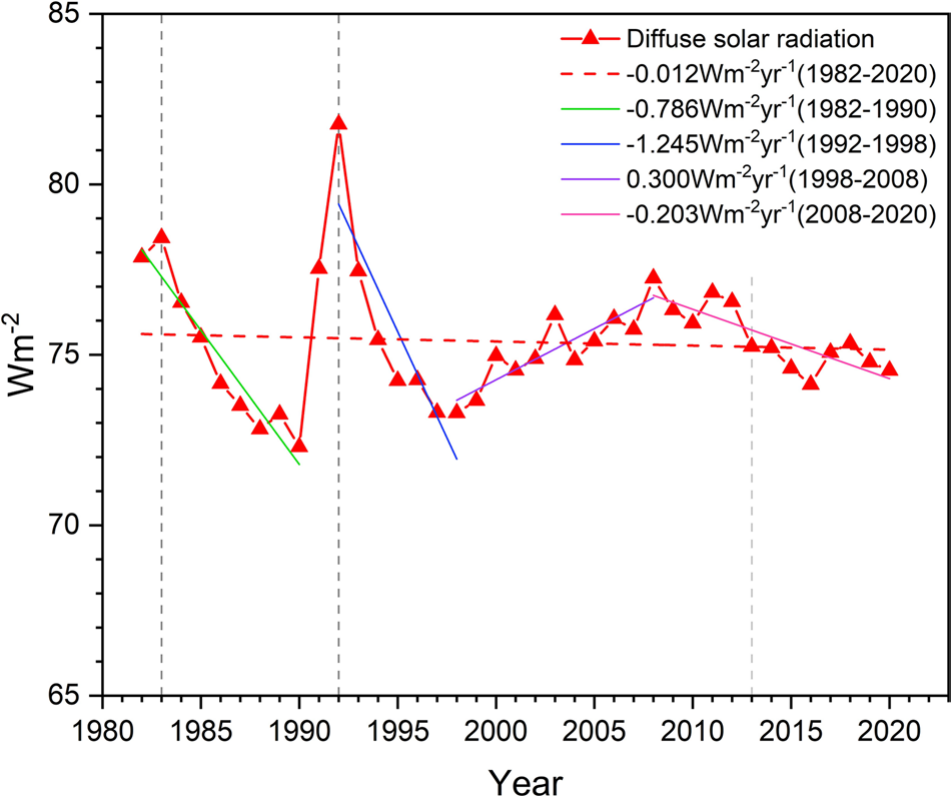
Interannual variation trends of DSR over China during 1982–2022.

Over China, the interannual trends of DSR from 1982 to 2020 are depicted in Fig. 8. Overall, the mean annual diffuse solar irradiation varied from 72.3 to 81.8 W m^−2^, exhibiting an overall decreasing trend of −0.012 W m^−2^ yr^−1^. More specifically, the figure delineates five periods with characteristic trends. From 1982 to 1990, the diffuse solar radiation showed a decreasing trend of −0.786 W m^−2^ yr^−1^. During 1992–1998, another downward period existed, with a mean trend of −1.245 W m^−2^ yr^−1^. An opposite trend followed in 1998–2008, characterized by a slight increase of 0.300 W m^−2^ yr^−1^. Finally, DSR was affected by a small decreasing tend of −0.203 W m^−2^ yr^−1^ during 2008–2020. These trends are purposedly not qualified as “dimming” or “brightening” to avoid confusion, since dimming relates to a decrease in GHI, which normally corresponds to an increase in DSR, and vice versa for brightening. Meanwhile, due to the lack of pre-2000 site data, the DSR trends before 2000 should be considered as indicative only. Regarding the question of the validity of the temporal variation of the dataset under long time series, in fact, according to the existing studies, the diffuse solar radiation exhibits a similar trend over the 39-year period as in the present study, which testifies to the validity of the model proposed in the present study until 2000^70–72^.

Remarkably, Fig. 8 displays two peaks in DSR, occurring in 1983 and 1992. Correspondingly, Fig. 7 shows that the national DSR widely peaked in 1983 and 1992. In 1983, the peak in DSR is directly related to a large global increase in aerosol concentration caused by the eruption of the EI Chichón volcano in Mexico, which resulted in an enhanced scattering effect^73^. Similarly, the eruption of Mount Pinatubo in the Philippines in 1991 caused a peak in DSR the following year (1992)^74^. Large amounts of volcanic aerosols were released into the stratosphere during those two eruptions, eventually resulting in a significantly additional burden in AOD, which affected the whole world for ≈2 years^75,76^. After 2000, rapid industrialization in Asia and an increase in sulphur-containing particle emissions led to an intensified atmospheric scattering effect^77^. This effect resulted in an annual increase in the distribution of DSR in Northern China as illustrated in Fig. 8, up to a peak that occurred in 2008. Subsequently, the observed decreasing trend in annual DSR can be attributed to the air quality measures and carbon emission reduction policies implemented by the Chinese government^78^.

### Uncertainty and limitations

**Fig. 9:**
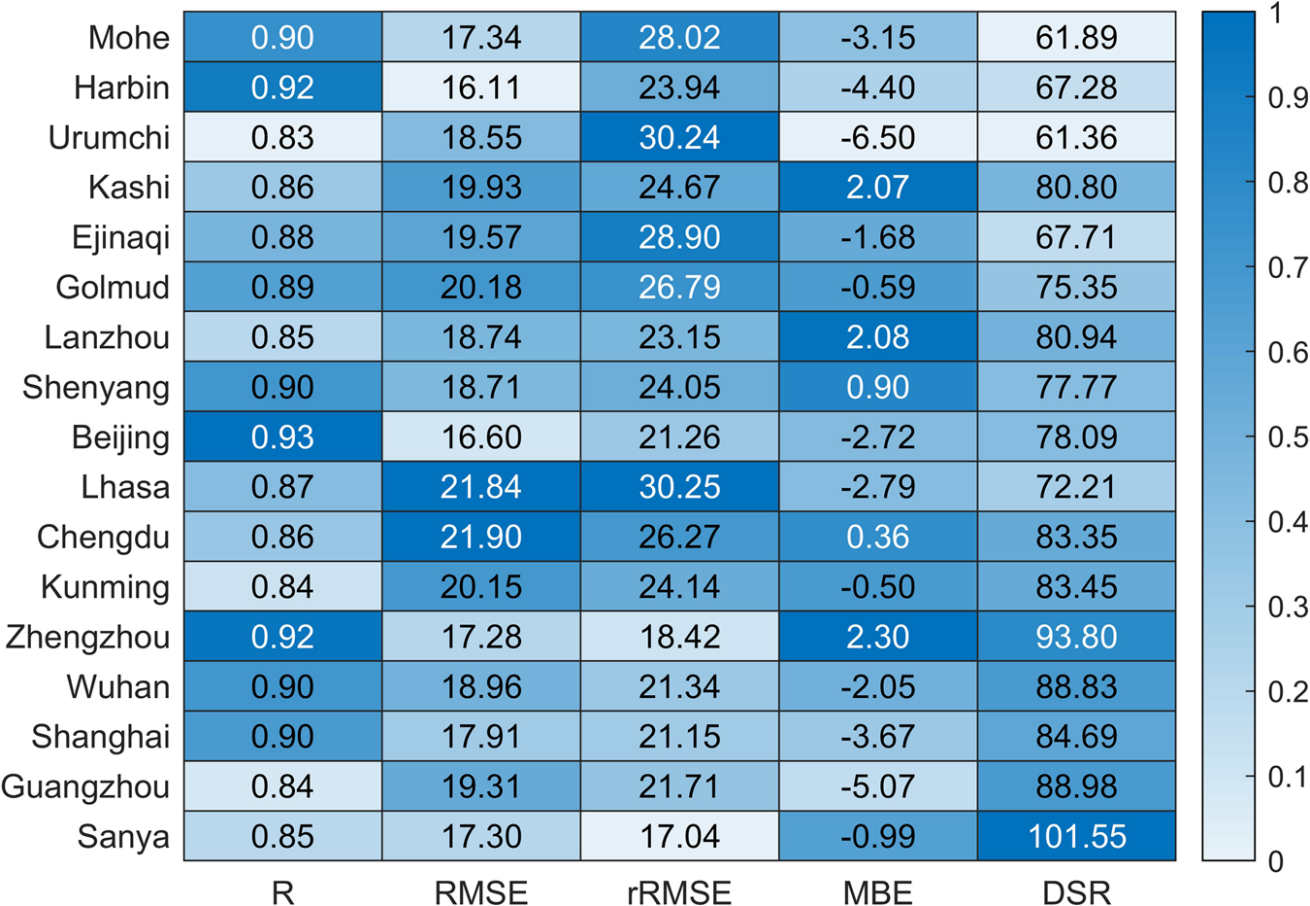
Heat map of accuracy results of DSR estimates at 17 DSR observations sites. Different color shades of each cell represent the size of the five accuracy metrics (R, RMSE, rRMSE, MBE, DSR).

Figure 9 depicts the validation results of the estimated DSR at the 17 test sites. Overall, the dataset maintains rRMSE under 30%, with over 70% of the stations displaying an RMSE below 20 W m^−2^. Moreover, more than 40% of the sites demonstrate a correlation coefficient greater than 0.9, while less than 19% of them exhibit a correlation coefficient between 0.85. These findings reflect a high agreement between the estimates and observations, particularly in the Northern (Beijing) and North-Eastern (Shenyang) parts of the country. Conversely, elevated RMSE values and diminished R values are predominantly observed in the Qinghai-Tibet Plateau and the Sichuan Basin — regions known for their dramatic weather changes and/or complex terrain. For those areas, the combination of spatial inhomogeneities in terrain and rapid spatiotemporal changes in cloud cover can directly affect the representativeness of the atmospheric inputs to the model, resulting in relatively larger deviations between modeled DSR and station observations. However, at stations with high levels of industrialization, such as Beijing, Shenyang, Wuhan or Shanghai, where atmospheric aerosols contain a lot of black carbon, the hybrid model yields good results, with R values exceeding 0.9 and RMSE below 20 W m^−2^. This suggests that the model is effective with regard to the impact of aerosols on DSR. In contrast, the model appears somewhat less accurate at Kunming (R = 0.84, RMSE = 20.15 W m^−2^, rRMSE = 24%), in other regions. That station is representative of Southwest China, where the average annual rainfall is much higher than elsewhere, thus indicative of larger and more complex cloudiness.

In conclusion, this study presents the most accurate dataset of diffuse solar radiation currently available for China, showing a consistently good correlation and homogeneity throughout mainland China. The dataset can serve as a fundamental resource for researchers investigating the long-term spatial distribution of diffuse solar radiation or other relevant studies. The study has identified several limitations, including reduced accuracy performance over areas affected by either tropical monsoon or terrain-included inhomogeneities, such as over the Qinghai-Tibet and Sichuan basin regions. Further refinements would be necessary to address the various possible causes of spatiotemporal variations in cloud cover. To that effect, and to better align the estimates with rapidly changing weather conditions, it is anticipated that the model can be improved by considering new inputs related to cloudiness, such as cloud height, wind speed, or temporal variability in cloud cover. Over China, the MERRA-2 and ERA5 datasets show significant differences in diffuse radiation, which may be partly due to other variables such as cloud cover^79^. Given the differences in aerosol data assimilation between MERRA-2 and ERA5, additional correction steps will need to be added to the model in the future to reduce the impact of inconsistencies from different data sources on the final results.

## Usage Notes

This developed diffuse solar radiation dataset (CHDSR) performs well when compared to the CMA stations during 2000–2015. However, the CHDSR could be affected by the lack of DSR observation results during the early years (1982–1990), the pre-2000 datasets are provided for information purposes only and need to be used with care for validation. Therefore, when using this dataset for early years, it should be considered only as a preliminary reference until further validation can be performed.

This long-term dataset is suitable for a better understanding of the spatial and temporal variations in diffuse solar radiation across China, and for further evaluations of photosynthetic efficiency.

## Data Availability

The complete code used in this work is not released along with the dataset because a part of it involves the REST2_v9.1 code, which cannot currently be shared without permission. Meanwhile, the readers could get the code from the home page of the REST2 model (https://www.solarconsultingservices.com/).
